# Superior Mesenteric Artery Syndrome: An Unusual Cause of Abdominal Pain

**DOI:** 10.7759/cureus.11505

**Published:** 2020-11-16

**Authors:** Sobia N Laique, Catherine F Vozzo, Prabhleen Chahal

**Affiliations:** 1 Gastroenterology, Mayo Clinic, Scottsdale, USA; 2 Gastroenterology, Cleveland Clinic, Cleveland, USA

**Keywords:** duodenum, chronic abdominal pain, superior mesenteric artery syndrome

## Abstract

Superior mesenteric artery (SMA) syndrome is caused by compression of the transverse duodenum at the angle between the aorta and the SMA that may lead to postprandial or constant epigastric pain, nausea, vomiting anorexia and weight loss. The diagnosis is often missed given nonspecific symptoms and low reported prevalence. The authors present a case of a 29-year-old female who presented with seven months of epigastric pain and significant weight loss. The patient was diagnosed with SMA syndrome with the aid of upper endoscopy, upper gastrointestinal barium study and computed tomography imaging.

## Introduction

Superior mesenteric artery (SMA) syndrome is an uncommon cause of duodenal obstruction. This occurs through compression of the third portion of the duodenum between the aorta and the SMA. The prevalence is reported to be 0.1%-0.3% and is more common in young women [1]. Risk factors include disorders predisposing to significant weight loss like anorexia nervosa or cancer, abdominal or spinal surgery such as spinal fusion, and anatomic abnormalities [2]. The typical clinical presentation includes postprandial epigastric pain, abdominal distension, weight loss, nausea and vomiting. Computed tomography and/or magnetic resonance angiography are generally used for diagnosis [2].

## Case presentation

A 29-year-old female presented to the outpatient gastroenterology clinic for evaluation of chronic upper abdominal pain over the past seven months. The pain was described as constant with intermittent worsening post-prandially. It was associated with nausea and vomiting after eating and constant bloating. She reported a gradual weight loss for seven months and then rapid weight loss of 20 pounds in the past three months secondary to increased severity of her symptoms. Physical exam was remarkable for body mass index of 17 (normal 18.5-24.9), generalized upper abdominal fullness, and normoactive bowel sounds. 

**Figure 1:**
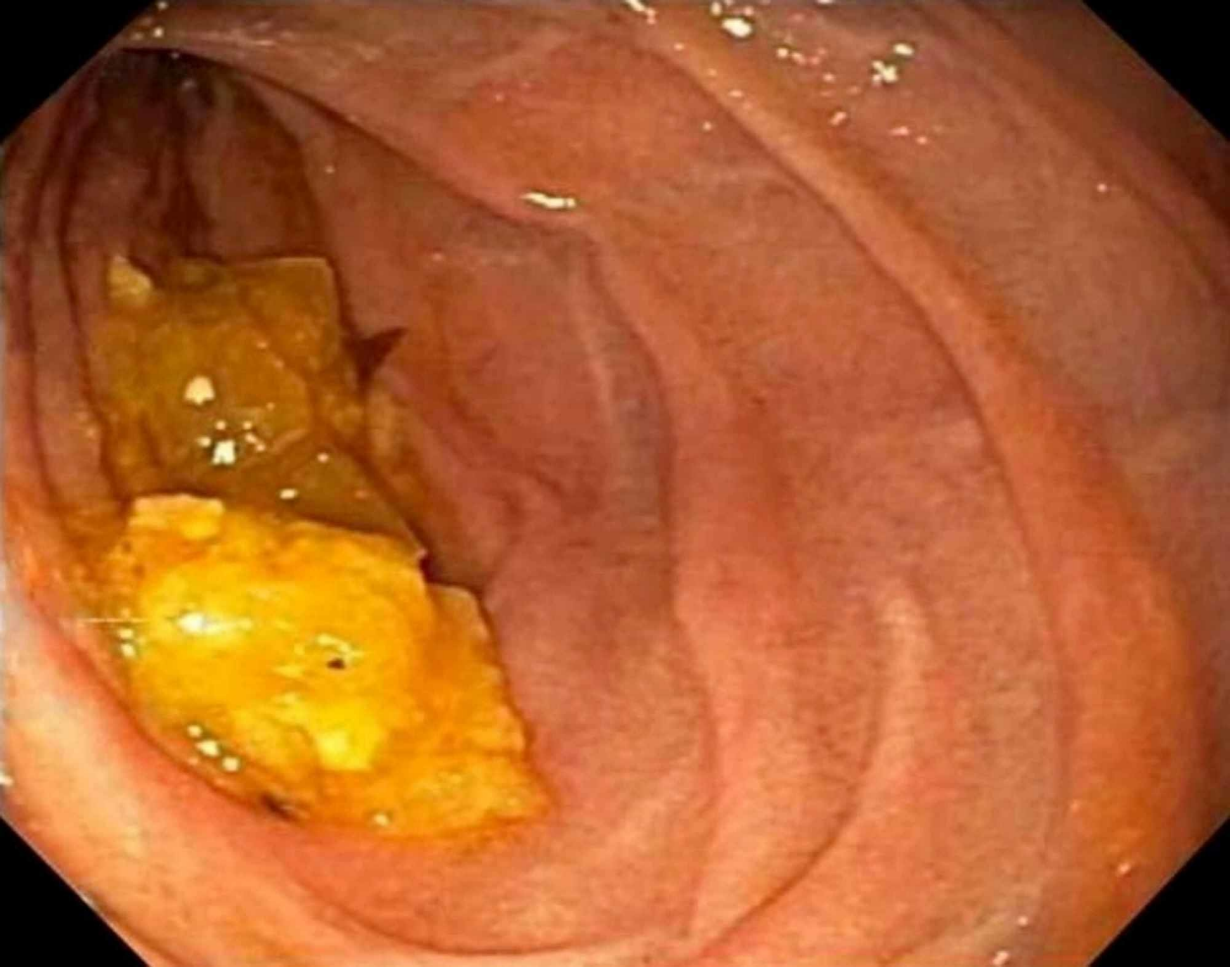
Esophagogastroduodenoscopy demonstrating an acquired, benign appearing extrinsic moderate stenosis in the third part of the duodenum

A recent outside hospital workup included a reportedly normal upper endoscopy, normal computed tomography scan of abdomen, and a normal gastric emptying study. Laboratory evaluation showed normocytic anemia with hemoglobin of 10.4 g/dL (normal 11.5-15.5 g/dL), calcium 8.9 mg/dL (normal 8.5-10.2 mg/dL), and albumin 3.0 g/dL (normal 3.9-4.9 g/dL) and normal liver biochemistry analysis. An upper endoscopy was repeated which showed food residue in the duodenal bulb and second portion of duodenum, dilation of the second portion of the duodenum, and an acquired, short, benign appearing extrinsic moderate stenosis in the third part of the duodenum (Figure 1).

**Figure 2:**
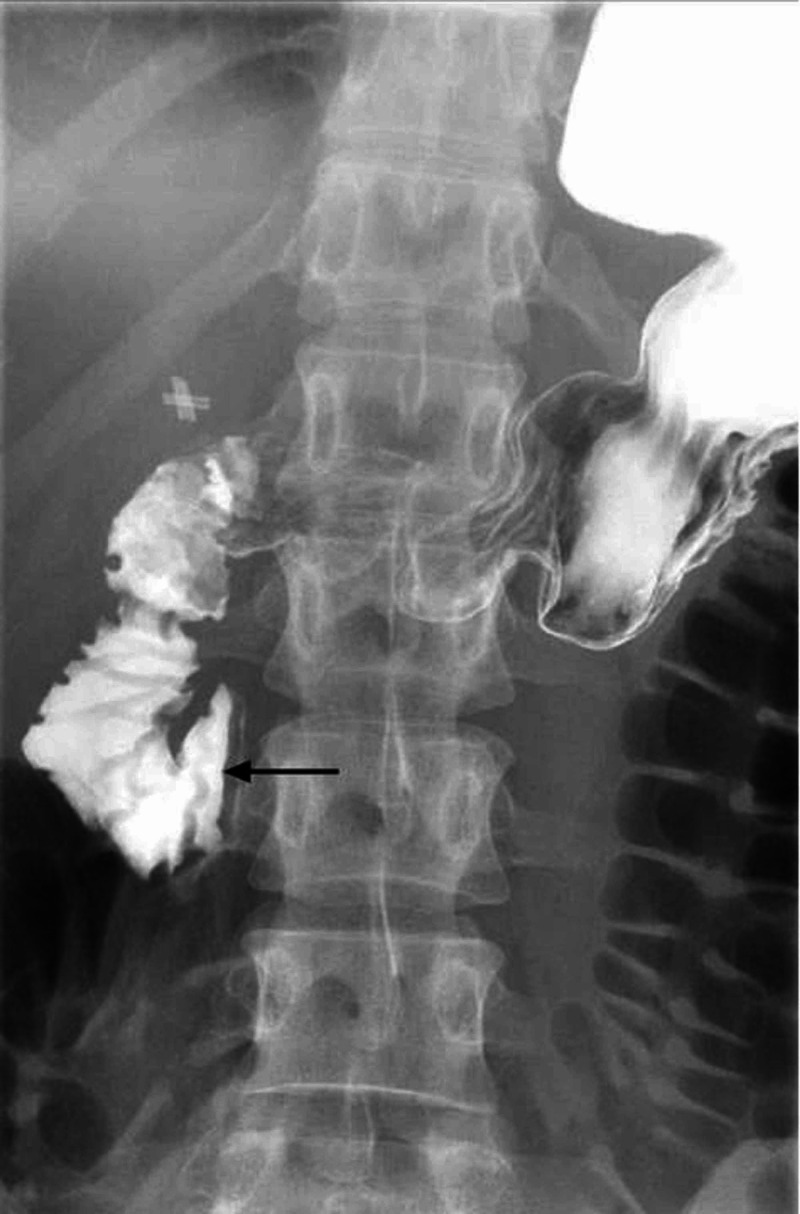
Upper gastrointestinal barium study with dilated second portion of duodenum with abrupt cutoff (arrow)

**Figure 3:**
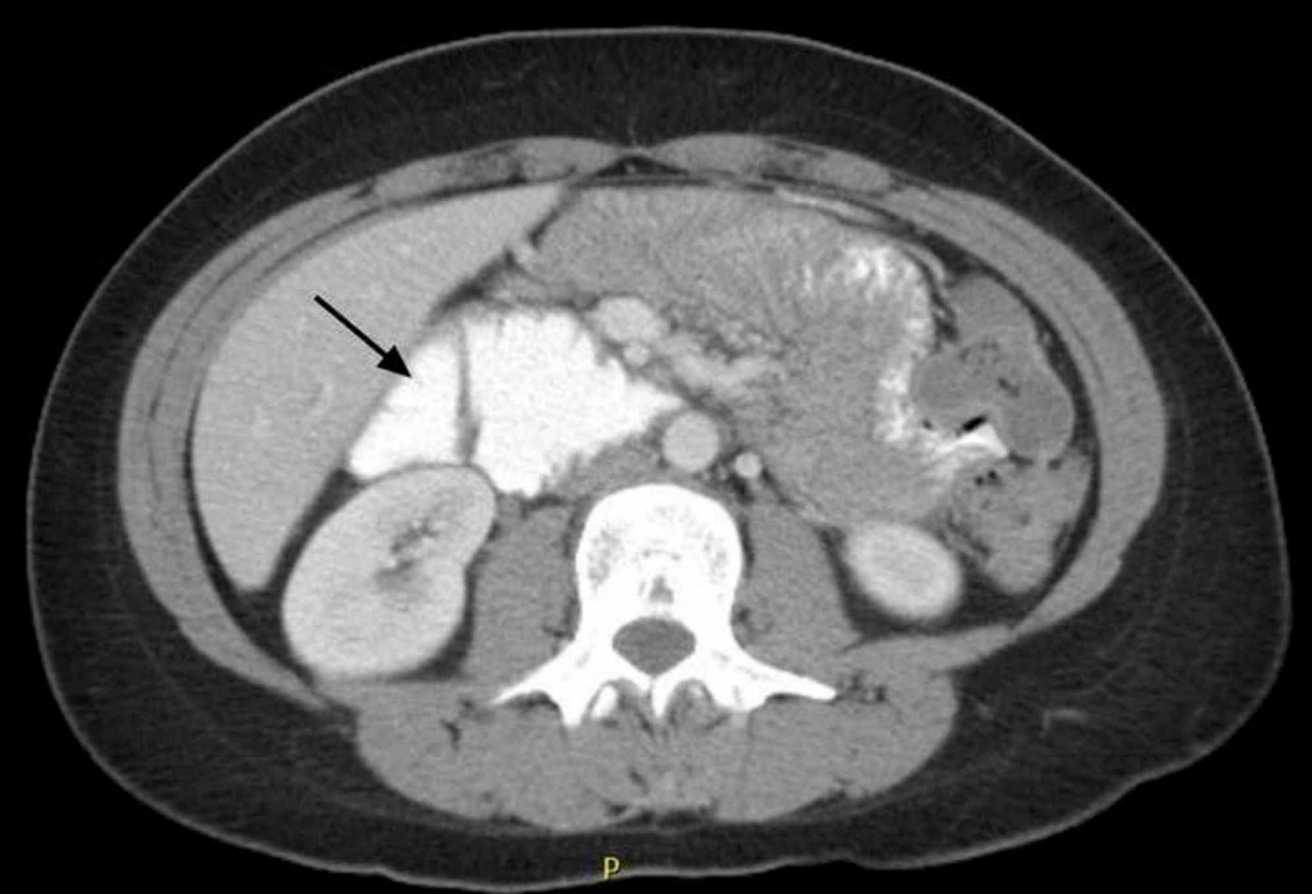
Coronal computed tomography image revealing dilated second portion of the duodenum (arrow)

**Figure 4:**
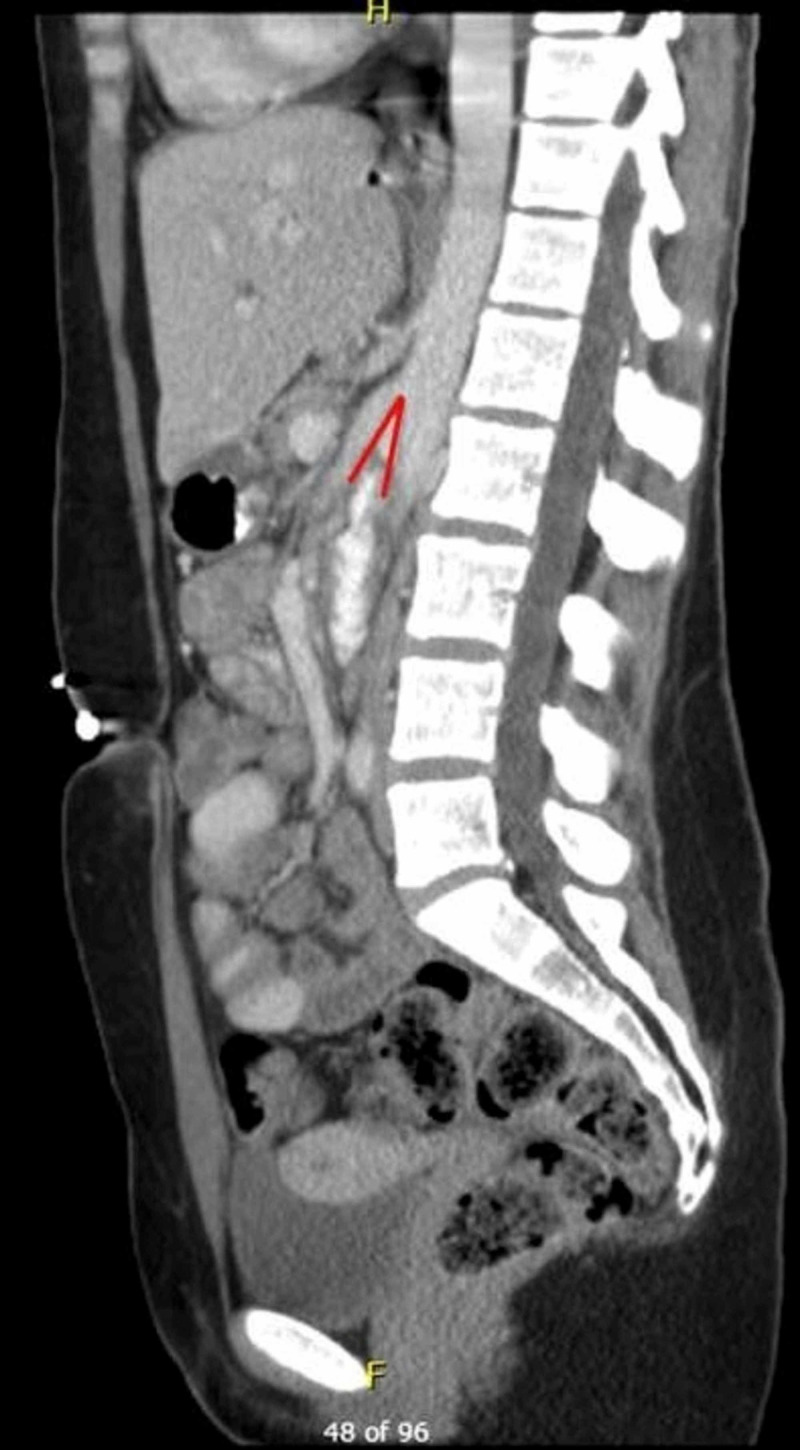
Sagittal computed tomography images reveal transverse duodenum compressed between the aorta and superior mesenteric artery The red bars reveal the aortosuperior mesenteric angle and demonstrate the aortomesenteric distance

Upper gastrointestinal barium study was performed and demonstrated dilated second and third part of the duodenum followed by an abrupt vertical extrinsic impression (Figure 2). Coronal computed tomography images, along with the aforementioned findings, showed the absence of retroperitoneal fat pad (Figure 3) with the sagittal images revealing the transverse duodenum compressed between the aorta and superior mesenteric artery. The aortomesenteric angle and aortomesenteric distance were 19° and 10 mm respectively, consistent with a diagnosis of SMA syndrome (Figure 4). The patient was referred for initiation of parenteral nutrition and vascular surgery consultation for possible Strong’s procedure. However, the patient declined surgery and received enteral nutrition via percutaneous jejunostomy tube. At three-month follow-up, her pain had improved and her weight had stabilized. 

## Discussion

SMA syndrome, first described in 1842 by Carl von Rokitansky, defines the constellation of symptoms resulting from compression of the transverse duodenum at the angle between the aorta and the superior mesenteric artery [3]. The narrowing is thought to be caused by loss of the intra-abdominal fat pad which serves to elevate the SMA away from the aorta to avoid duodenal obstruction [3]. Patients may present with nonspecific findings in either the acute or chronic setting and a high index of suspicion is required to make the diagnosis which is often delayed or missed. Diagnosis relies on the correlation of clinical symptoms and radiographic findings.

In the presented case, SMA syndrome was first suspected due to esophagogastroduodenoscopy (EGD) findings of retained food in the duodenum, a dilated second portion of the duodenum and an apparent narrowing of the third part of the duodenum, with no clear obstructing mass. EGD, while commonly performed in patients ultimately diagnosed with SMA syndrome, rarely have such pronounced obstructive findings (food retention with distal duodenal narrowing and upstream dilation) [3]. More common EGD findings include esophagitis, gastritis, or duodenitis caused by duodenogastric reflux [2]. 

In clinical practice, a combination of upper gastrointestinal barium study, contrast-enhanced CT, MR angiography and/or upper endoscopy may be used for diagnosis. In general we recommend upper endoscopy to rule out alternative pathology (mass obstructing lesion), barium swallow, followed by CT versus MR angiography. Radiologic features utilized to diagnose SMA syndrome include duodenal obstruction with an abrupt cutoff in the third portion of the duodenum on gastrointestinal barium study and an aortosuperior mesenteric angle of less than 25° on CT or MR angiography, particularly when in association with a aortomesenteric distance of less than 8 mm [3-5].

Initial treatment is conservative for most cases of SMA syndrome and includes gastrointestinal decompression with nutritional support. Although there is no clear guideline on when surgical management should be pursued, more recent recommendations suggest not exceeding three months of conservative management [2]. Surgical options include gastrojejunostomy, duodenojejunostomy, and Strong’s procedure (duodenal mobilization for lowering the duodenojejunal flexure) [6]. Current data in the literature do not support one surgical procedure over another as no randomized trials are available [2]. 

## Conclusions

SMA syndrome is a rare condition that presents as a challenging diagnosis given the nonspecific nature of the presentation. Clinicians should have a high index of suspicion in cases where risk factors such as extreme weight loss, anatomical abnormalities, or prior abdominal/spinal surgeries are present. The diagnostic algorithm in suspected cases of SMA syndrome should include EGD, upper gastrointestinal barium study, and contrast-enhanced CT or MR angiography. This case is unique as it highlights the classic findings of SMA syndrome seen in each of these diagnostic modalities. Furthermore, EGD does not lead to the diagnosis of SMA syndrome, but rules out alternative causes such as GI luminal pathology that may mimic SMA syndrome. In this rare case, the patient's endoscopic findings ultimately led to the formal diagnosis of SMA syndrome, revealing the importance of a keen and thoughtful eye during the endoscopic evaluation of patients with risk factors.
